# Identifying signals associated with psychiatric illness utilizing language and images posted to Facebook

**DOI:** 10.1038/s41537-020-00125-0

**Published:** 2020-12-03

**Authors:** Michael L. Birnbaum, Raquel Norel, Anna Van Meter, Asra F. Ali, Elizabeth Arenare, Elif Eyigoz, Carla Agurto, Nicole Germano, John M. Kane, Guillermo A. Cecchi

**Affiliations:** 1 The Zucker Hillside Hospital, Northwell Health, Glen Oaks, NY USA; 2 The Feinstein Institute for Medical Research, Manhasset, NY USA; 3 The Donald and Barbara Zucker School of Medicine at Hofstra/Northwell, Hempstead, NY USA; 4 IBM Research, Thomas J. Watson Research Center, Yorktown Heights, NY USA

**Keywords:** Schizophrenia, Psychosis

## Abstract

Prior research has identified associations between social media activity and psychiatric diagnoses; however, diagnoses are rarely clinically confirmed. Toward the goal of applying novel approaches to improve outcomes, research using real patient data is necessary. We collected 3,404,959 Facebook messages and 142,390 images across 223 participants (mean age = 23.7; 41.7% male) with schizophrenia spectrum disorders (SSD), mood disorders (MD), and healthy volunteers (HV). We analyzed features uploaded up to 18 months before the first hospitalization using machine learning and built classifiers that distinguished SSD and MD from HV, and SSD from MD. Classification achieved AUC of 0.77 (HV vs. MD), 0.76 (HV vs. SSD), and 0.72 (SSD vs. MD). SSD used more (*P* < 0.01) perception words (hear, see, feel) than MD or HV. SSD and MD used more (*P* < 0.01) swear words compared to HV. SSD were more likely to express negative emotions compared to HV (*P* < 0.01). MD used more words related to biological processes (blood/pain) compared to HV (*P* < 0.01). The height and width of photos posted by SSD and MD were smaller (*P* < 0.01) than HV. MD photos contained more blues and less yellows (*P* < 0.01). Closer to hospitalization, use of punctuation increased (SSD vs HV), use of negative emotion words increased (MD vs. HV), and use of swear words increased (*P* < 0.01) for SSD and MD compared to HV. Machine-learning algorithms are capable of differentiating SSD and MD using Facebook activity alone over a year in advance of hospitalization. Integrating Facebook data with clinical information could one day serve to inform clinical decision-making.

## Introduction

Mental illness occurs in ~20% of the population worldwide^1,2^ and can be associated with a significant individual, familial, and societal burden^3–5^. Psychiatric symptoms often emerge during the formative years of adolescent and young adult development and interfere with the establishment of healthy social, educational, and vocational foundations^6^. While early intervention services have demonstrated the potential to improve outcomes^7,8^, symptoms often remain untreated for months or even years before receiving clinical attention^9^. Identifying and engaging individuals with psychiatric illness earlier remains a major public health challenge^3^. Novel strategies, supported by technological innovation, are critical to achieving this goal.

A growing body of literature describes linguistic and behavioral patterns, extracted from social media sites like Facebook and Twitter, that are associated with psychiatric diagnoses and symptoms^10–19^. This research aims to inform the development of digital tools to assist in the identification and monitoring of individuals with mental illness. Adolescents and young adults^20–24^ are both the highest utilizers of social media and among those at the highest risk for the development of a psychiatric illness^7^. Consequently, social media activity has the potential to serve as a rich source of objective and clinically meaningful collateral information.

Research on the relationship between social media activity and behavioral health is promising. Personalized patterns of social media use have predicted personality traits, demographic characteristics, intelligence, substance use, and religious and political views^25,26^. Reports have found that Facebook status updates reveal active symptoms of depression in college students^10^, predict a diagnosis of depression in those presenting to the emergency room^11^, and predict symptomatic exacerbations in individuals with schizophrenia spectrum disorders (SSD)^19^. Additionally, changes in language use on Twitter and Reddit have been linked to the onset of a depressive episode^12^ and post-partum mood disorder^16^, and have been used to predict diagnoses of post-traumatic stress disorder^13^, bipolar disorder^27^, and schizophrenia^18^, as well as binge drinking^17^, and suicidal ideation^14^ with high degrees of accuracy.

The majority of studies to date have made assumptions about clinical and diagnostic status. Most have utilized a computational approach to flag publicly available social media data from profiles of users who self-disclose having a diagnosis^27–30^ without the ability to assess whether the individual meets diagnostic criteria for a psychiatric disorder. Other studies have extracted user data from anonymous individuals affiliated with an online mental health support group^31–33^, or have relied on clinicians to attempt to appraise the validity of a self-reported diagnoses^18,34,35^. Studies that link social media activity to clinically confirmed psychiatric symptoms or diagnoses are rare^11,18^, and without confirmed diagnoses, the clinical utility of findings is limited^36^.

Toward the goal of applying novel, social media-based approaches to identify and monitor individuals in need of psychiatric care, research using real patient data with clinically confirmed diagnoses is necessary. The success of predictive algorithms is entirely dependent on access to sufficient high-quality patient data^36^. We, therefore, aimed to explore linguistic and behavioral signals associated with psychotic and mood disorders in Facebook activity archives collected from consented patients receiving psychiatric care. We hypothesized that language use and image choice would differentiate individuals with SSD, mood disorders (MD), and healthy volunteers (HV). We additionally hypothesized that differences would be greater closer to the date of the first psychiatric hospitalization, consistent with escalating psychiatric symptoms experienced during this time.

## Results

A total of 3,404,959 Facebook messages and 142,390 Facebook Images across 223 participants (mean age = 23.7 years; 41.7% male) with SSD (*n* = 79), MD (*n* = 74), and HV (*n* = 70) were collected (Tables 1 and 2).

**Table 1:** Participant demographics.

	SSD	MD	HV	Full sample
*N*	79	74	70	223
	Mean (SD)			
Age	23.9 (4.17)	22.01 (3.72)	25.34 (5.53)	23.72 (4.69)
	*N* (%)			
Male	53 (67.09)	20 (27.03)	20 (28.57)	93 (41.70)
*Race/ethnicity*				
African American/Black	39 (49.36)	16 (21.62)	10 (14.28)	65 (29.14)
Asian	12 (15.19)	14 (18.92)	13 (18.57)	39 (17.48)
Caucasian	19 (24.05)	31 (41.89)	44 (62.86)	94 (42.15)
Mixed race/other	9 (11.39)	13 (17.56)	3 (4.28)	25 (11.21)
Hispanic	12 (15.19)	12 (16.22)	2 (2.86)	26 (11.65)
*Diagnosis*				
Schizophrenia	39 (49.37)	–	–	39 (49.37)
Schizophreniform	15 (18.99)	–	–	15 (18.99)
Schizoaffective	14 (17.72)	–	–	14 (17.72)
Unspecified SSD	11 (13.92)	–	–	11 (13.92)
Bipolar disorder (manic episode)	–	8 (10.81)	–	8 (10.81)
Bipolar disorder (depressed episode)	–	2 (2.70)	–	2 (2.70)
Bipolar disorder (mixed episode)	–	2 (2.70)	–	2 (2.70)
Major depressive disorder	–	62 (83.78)	–	62 (83.78)

**Table 2:** Number of participants with Facebook messenger and image data per trimester per group.

Group	Trimester	Number of subjects with messages	Number of subjects with images	Number of average messages (s.d.)	Number of average word count (s.d.)	Number of average images (s.d.)
HV	1T	66	32	489 (1042)	3546 (6379)	22 (56)
	2T	69	24	566 (1231)	4068 (7967)	9 (11)
	3T	67	25	517 (1238)	3384 (7293)	80 (282)
	4T	64	26	609 (1379)	4151 (8932)	98 (368)
	5T	62	27	601 (1222)	3993 (7679)	99 (377)
	6T	58	26	672 (1183)	4504 (6979)	43 (73)
MD	1T	56	39	680 (953)	4895 (7609)	33 (81)
	2T	63	37	653 (1139)	4201 (6125)	25 (83)
	3T	62	40	752 (1260)	4497 (7050)	16 (33)
	4T	57	37	737 (1220)	4509 (6109)	37 (89)
	5T	56	38	663 (973)	4559 (6036)	27 (77)
	6T	52	34	725 (1082)	4090 (5734)	72 (226)
SSD	1T	59	35	865 (2117)	4796 (9182)	3 (2)
	2T	57	30	690 (1747)	3808 (7559)	17 (60)
	3T	55	31	766 (2245)	4009 (8599)	63 (314)
	4T	56	26	601 (1134)	3926 (6965)	11 (39)
	5T	57	32	569 (934)	4055 (5895)	10 (21)
	6T	53	33	552 (836)	3874 (6081)	12 (30)

### Machine-learning models

**Fig. 1:**
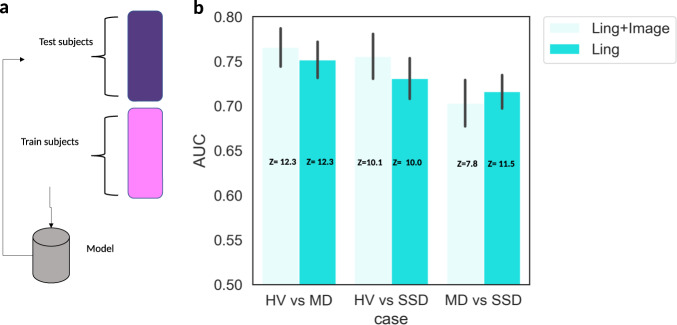
Classification performance for binary classification case, using fivefold (by the participant) cross-validation. **a** shows data separation in train and test set. **b** Bars show mean of 50 runs, vertical lines denote standard deviation. Light cyan bars show results using all features, cyan bars show results when using only linguistic features.

Figure 1 represents the average area under the curve (AUC) using data from all 18 months before hospitalization (consent date for HV). Comparing HV to MD, classification performance achieved an AUC of 0.77 when integrating both image and linguistic features and an AUC of 0.76 when comparing HV to SSD. Comparing SSD to MD, the model achieved an AUC of 0.72 using linguistic features alone, and performance was reduced slightly to 0.70 by incorporating image data. Additional experimentation (not reported) showed that alternative approaches to Random Forest for feature aggregation did not yield improvements. We hypothesize that this is not due to an intrinsic interference of the image features but rather due to insufficient participants to match the number of features considered. All results surpass chance probability. The *Z* scores included in Fig. 1 show the number of standard deviations above chance for each classification. *P* values for classification are all *P* < 0.001.

To explore if the time to hospitalization had an effect on classification, we used data from nonoverlapping trimesters to compute the features. To ensure that we detect true differences in psychiatric conditions, and are not only recognizing features unique to a particular participant, but we also trained our classification model on data from the first trimester, closest to the date of hospitalization, and then tested the model on the remaining participants, across all six trimesters (transferring the model from one dataset to another).

**Fig. 2:**
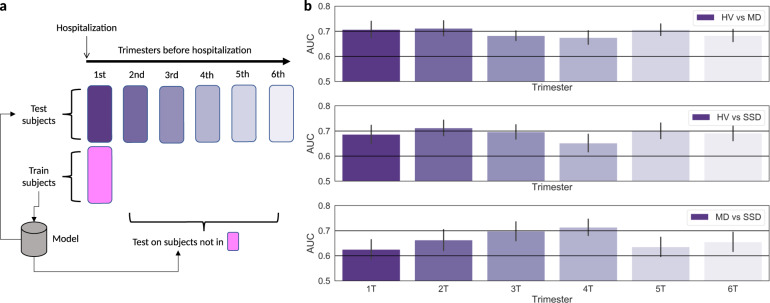
Classification performance for binary classification for each trimester. **a** shows data separation in train and test set. **b** Bars show mean of 50 runs, vertical lines denote standard deviation. Top of panel **b** show results for HV vs MD classification; the middle bars show results for HV vs. SDD classification; bottom bars show results for schizophrenia spectrum disorders (SSD) vs. MD.

Figure 2 represents the average AUC for each trimester. For HV compared to MD, the AUC varied from 0.71 in the first trimester to 0.68 in the sixth trimester, remaining mostly stable. In the fifth trimester, over a year in advance of the first psychiatric hospitalization, the AUC reached 0.71. For HV compared to SSD, the AUC was 0.69 in the first and sixth trimesters, reached an AUC of 0.71 in the second trimester, and dipped to 0.65 in the fourth trimester. In the fifth trimester, over a year in advance of the first psychiatric hospitalization, the AUC reached 0.70. When comparing SSD to MD the average AUC reached its best performance of 0.71 in the fourth trimester, a year in advance of hospitalization. All classification results surpass chance probability.

Table 3 demonstrates the performance metrics for the three-way classification. The algorithms correctly classified participants with SSD from those with MD or HV with an accuracy of 52% (chance = 33%). Participants with MD were correctly classified with an accuracy of 57% (chance = 37%), and HVs were correctly classified with an accuracy of 56% (chance = 29%). We further conducted an error analysis by accessing data from medical records to understand the specific instances of repeatedly mislabeled data/incorrect predictions (participants misclassified at least 80% of the time, *n* = 22). Half (*n* = 11, 22%) were either SDD confused with MD (*n* = 8) or MD confused with SSD (*n* = 3). For each of these instances, two co-authors reviewed the accompanying medical records. In seven (88%) of the participants with SSD misclassified as MD, significant mood symptoms had been documented by the treatment team. Similarly, two (67%) of participants with MD misclassified as SSD experienced auditory hallucinations as per the medical records.

**Table 3:** Performance metrics for the three-way classification.

Group	Accuracy	F1	Chance
SSD	0.52	0.53	0.33
MD	0.57	0.57	0.37
HV	0.56	0.54	0.29

### Image features

**Fig. 3:**
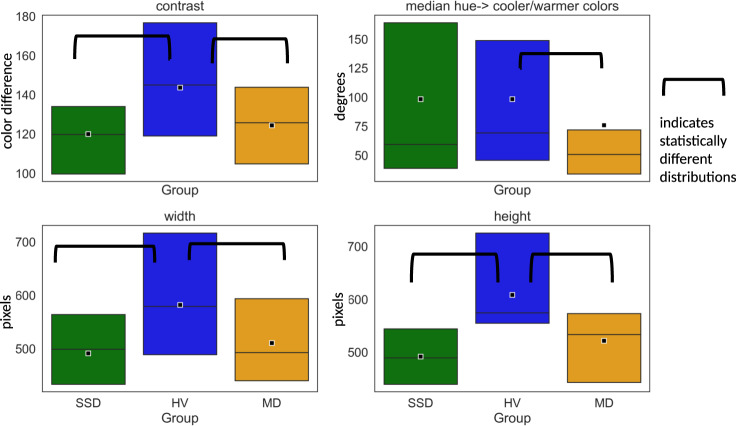
Boxplots showing significantly different feature distributions from images posted to Facebook. The box represents data from 25th quartile to 75th quartile; quartile 50th (median) is indicated with a horizontal line; the mean (average) is represented with a square in the box.

Compared to HV, both height and width of photos posted by SSD or MD, were significantly smaller (*P* < 0.01). Participants with MD posted photos with more blue and less yellow colors, measured with median hue, relative to HV (*P* < 0.01) (see Fig. 3).

### Language features

**Fig. 4:**
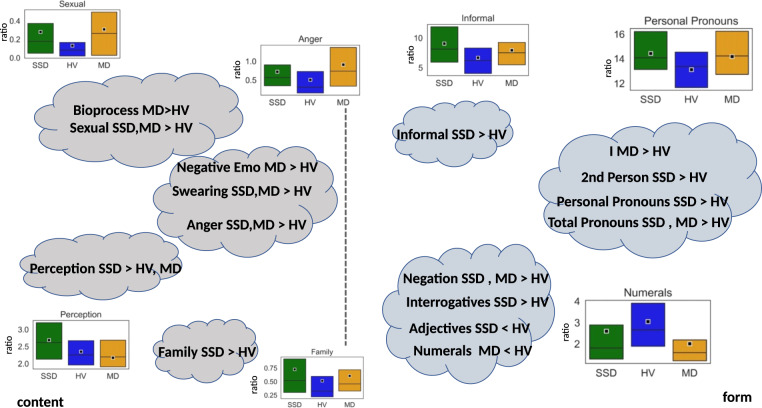
Statistically significant differences in linguistic features among groups depicted in a conceptual grouping. Boxplots show distributions for the three classes for a representative case of each “cloud”.

**Fig. 5:**
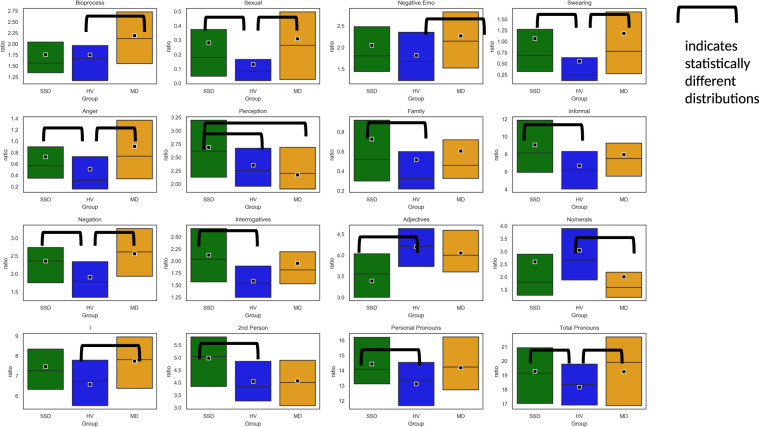
Boxplots showing statistically significant different feature distributions. The box represents data from 25th quartile to 75th quartile; quartile 50th (median) is indicated with a horizontal line; the mean (average) is represented with a square in the box.

Participants with SSD were significantly more likely to use words related to perception (hear, see, feel) compared to MD or HV (*P* < 0.01). Participants with both SSD and MD were significantly more likely to use swear words and anger-related language (*P* < 0.01) compared to HV. Participants with SSD were significantly less likely to use periods and punctuation marks (*P* < 0.01) compared to HV. Participants with SSD were significantly more likely to express negative emotions compared to HV, as well as use second person pronouns (*P* < 0.01). Words related to biological processes (blood, pain) as well as first person pronouns were used more often by MD compared to HV (*P* < 0.01). Informal language, in the form of netspeak (btw, lol, thx), was used significantly more by SSD compared to HV (*P* < 0.01). The use of pronouns and negations were significantly lower in HV compared to both SSD and MD (*P* < 0.01) (see Figs. 4 and 5 below and the complete list in Table 4). Age, sex, and race were not associated with linguistic differences in SSD or HV participants. Males with MD were significantly more likely (*P* < 0.01) to use numerals compared to MD females.

**Table 4:** Kolmogorov–Smirnov (KS) test results, significance, and effect sizes for features that are statistically significant in at least one of the three comparisons.

	HV vs. MD			HV vs. SSD			MD vs. SSD		
LIWC feature	KS	*P* value	Cohen’s d	KS	*P* value	Cohen’s d	KS	*P* value	Cohen’s d
Total pronouns	**0.26**	**3.01E-12**	0.10	**0.24**	**2.50E-10**	0.03	0.08	1.77E-01	0.07
Personal pronouns	0.23	4.47E-09	0.16	**0.23**	**1.57E-09**	0.15	0.05	6.39E-01	0.00
First person singular	**0.25**	**1.09E-10**	0.26	0.17	1.32E-05	0.17	0.08	1.61E-01	0.09
Second person	0.09	9.55E-02	0.04	**0.24**	**6.08E-10**	0.28	0.20	3.39E-07	0.31
Negation	**0.35**	**4.26E-21**	0.53	**0.23**	**3.41E-09**	0.23	0.14	7.20E-04	0.27
Adjectives	0.09	1.03E-01	0.04	**0.21**	**3.30E-08**	0.22	0.16	1.41E-04	0.17
Interrogatives	0.20	7.25E-07	0.16	**0.32**	**1.78E-15**	0.32	0.17	3.74E-05	0.17
Numerals	**0.23**	**4.66E-09**	0.53	0.15	2.30E-04	0.08	0.11	1.68E-02	0.27
Negative emotions	**0.26**	**3.42E-12**	0.41	0.14	8.70E-04	0.09	0.15	2.03E-04	0.27
Anger	**0.30**	**0.00E** + **00**	0.62	**0.19**	**1.90E-06**	0.28	0.13	4.21E-03	0.36
Family	0.17	2.70E-05	0.05	**0.22**	**7.88E-09**	0.15	0.12	7.53E-03	0.25
Perception	0.07	2.27E-01	0.00	**0.15**	**2.17E-04**	0.08	**0.13**	**2.24E-03**	0.05
Biological process	**0.24**	**2.69E-10**	0.29	0.10	3.89E-02	0.11	0.18	4.71E-06	0.42
Sexual	**0.27**	**3.65E-13**	0.54	**0.19**	**7.50E-07**	0.32	0.13	3.75E-03	0.14
Informal	0.23	2.53E-09	0.28	**0.33**	**1.78E-15**	0.42	0.14	8.75E-04	0.15
Swearing	**0.31**	**5.54E-16**	0.58	**0.20**	**4.51E-07**	0.33	0.12	6.95E-03	0.25

Cases that pass FDR correction (alpha = 0.01) are shown in bold.

**Fig. 6:**
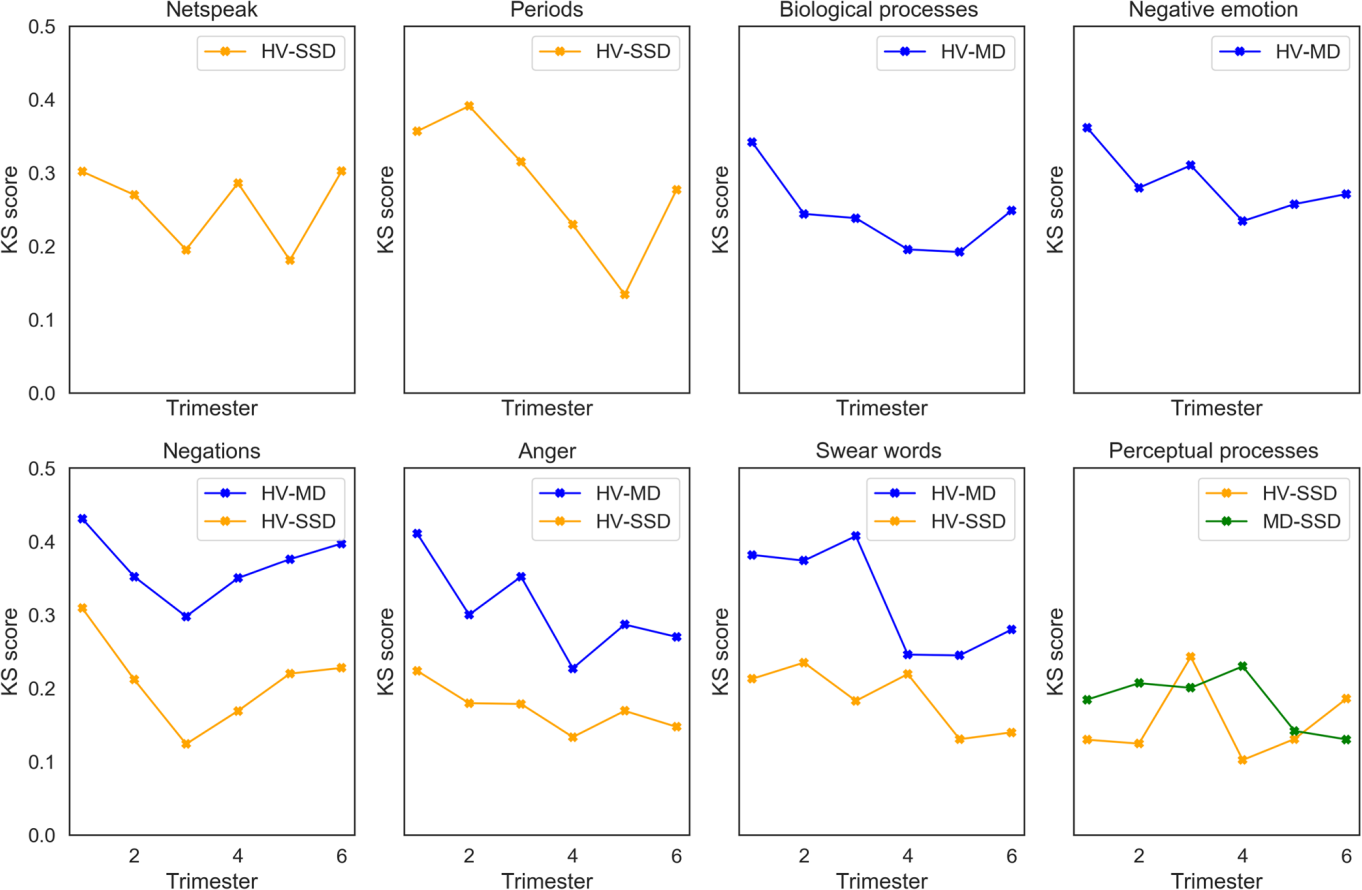
Statistically significant differences among groups as a function of time. Kolmogorov–Smirnov (KS) score demonstrating the trend in differences among groups as a function of time (distance to hospitalization date). Each panel represents a single feature.

Next, we assessed features as a function of time, to explore changes in Facebook activity that might accompany escalating symptoms, closer to the date of the first psychiatric hospitalization. As seen in Fig. 6, netspeak and use of periods become increasingly different between HV and SSD closer to the date of hospitalization. Similarly, when comparing HV to MD, differences in the use of words related to biological processes (blood, pain) and use of words related to negative emotions increased closer to the hospitalization date. Differences in the use of negations increase for both SSD and MD compared to HV. Differences in the use of anger-oriented language and swear words became greater closer to the date of hospitalization for SSD and MD compared to HV.

## Discussion

This project aimed to differentiate individuals with SSD, MD, and HV based on linguistic and image data uploaded to Facebook directly from participants with clinically confirmed psychiatric diagnoses, and to integrate clinical data obtained from the medical records. Results demonstrate that machine-learning algorithms are capable of identifying those with SSD and MD using Facebook activity alone over a year in advance of the first psychiatric hospitalization. For some features, differences among the populations were more pronounced closer to the date of the first hospitalization, consistent with escalating psychiatric symptom severity observed during this time. While Facebook alone is not meant to diagnose psychiatric conditions or to replace the critical role of a clinician in psychiatric assessment, our results suggest that social media data could potentially be used in conjunction with clinician information to support clinical decision-making. Much like an X-ray or blood test is used to inform health status, Facebook data, and the insights we gather, could one day serve to provide additional collateral, clinically meaningful patient information.

Compared to HV, participants with SSD and MD demonstrated significant differences in the use of words related to “anger”, “swearing”, “negative emotions”, “sex”, and “perception”. Several linguistic differences existed well in advance of the first hospitalization, suggesting that certain linguistic features may represent a trait rather than a state marker of psychiatric illness, or that subtle clinically meaningful changes are occurring (and manifesting online) long before hospitalization, as is often the case with SSD^37^. Analyzing word choice on Facebook could potentially help clinicians identify people at high risk of SSD or MD prior to the emergence of clinically significant symptoms. Furthermore, the use of several word categories became increasingly different closer to the date of hospitalization, likely reflecting changes in anxiety, mood, preoccupations, perceptions, social functioning, and other domains known to accompany illness emergence^37–39^. Understanding linguistic differences and incremental changes on Facebook could create important opportunities for intervention.

Prior work in speech and text analysis has identified linguistic markers associated with SSD and MD including significant differences in word frequency, word categories, as well as sentence coherence and structure^40–55^. Language analysis is now being used to classify mental health conditions based on text uploaded to social media platforms^10–15,17,18,25,26,56–58^. While this is a significant step forward, social media-based communication is unique due to short sentence structure, use of abbreviations, and distinct writing style. Our project extends this work by using language data extracted from the Facebook messenger, which represents a particularly unique data source, as prior research has nearly exclusively relied on Facebook status updates and/or Twitter Tweets. In contrast to status updates or Tweets, which are typically geared towards a larger audience, messenger represents private communication between two or more individuals and likely characterizes a more unedited and unfiltered form of text-based communication. As we continue to build language-based classifiers, the source of the language data must be taken into consideration. Moreover, to improve generalizability and clinical utility, future projects should identify which linguistic features might be independent of context, and which are relevant only to a specific medium.

Images uploaded varied across various metrics including photo size, contrast, and color. Although we are unable to exclude the potential impact of differing phone/camera quality, identified features may represent changes in the way participants with SSD and MD chose to express themselves online through image selection on Facebook. This may include differences in the way images are cropped (impacting height and width), and filter choice (impacting the contrast and colors). Prior work in image analyses suggests that photo and color preferences can be associated with personality characteristics^59–62^. In addition, studies have suggested that healthy participants tend to prefer brighter, more vivid colors while participants with depression prefer darker, grayer colors^63,64^. In line with prior work, we found that photos posted by participants with mood disorders tended to be bluer^65^. Participants with SSD have reported a preference for the color green^66,67^ though this has not been consistently found in the literature and was not supported by our findings^68^.

Our classifier achieved an AUC of over 0.75 when comparing participants with SSD or MD to HV and achieved an AUC of 0.72 when comparing those with SSD to MD using data uploaded to Facebook 18 months in advance of the first psychiatric hospitalization. In contrast to expectations, classification performance did not improve when utilizing Facebook data from only the first trimester, closest to the first psychiatric hospitalization, when psychiatric symptoms are typically the most prominent. This is likely due to the corresponding reduction in the overall quantity of available data to build the models. Performance remained relatively consistent throughout the trimesters suggesting that some social media-based markers might be present over a year in advance of the hospitalization date. Findings suggest that Facebook data may be capable of identifying early warning signs of emerging mental illness, or perhaps risk factors associated with the later development of mental illness. Future research will need to explore precisely when changes first manifest online as well as their clinical significance. Furthermore, although Facebook data alone cannot yet be used to make a diagnosis, the integration of this information with clinical data could help to improve the accuracy and reliability of clinical diagnoses^69,70^. Alternatively, the classifier could serve as a low burden screening tool for youth at risk of developing psychiatric disorders, providing objective collateral information, and identifying those in need of further evaluation. Additional research is required to explore the feasibility and acceptability of incorporating such a tool into existing screening procedures.

There are several noteworthy limitations to this study. First, some participants were more active on Facebook than others, providing varying degrees of data. An important question for future research will be how much social media data is necessary to make a reliable clinical prediction. Second, Facebook archives used for our analyses were collected retrospectively. While retrospective collection eliminates the possibility of altering behavior as a result of being monitored^71^, to achieve the goal of early symptom identification, prospective monitoring will be necessary. Third, eligibility criteria ranged from 15 to 35 years to reflect the inclusion criteria of the Early Treatment Program, however, adolescents may engage with social media in a distinct manner compared to young adults and will need to be considered in future initiatives. Fourth, the scope of our analyses was limited to Facebook messenger and photos. While this represents a significant portion of an individuals’ Facebook data, it is possible that findings would be different had additional components been included. Fifth, both individuals with bipolar disorder as well as individuals with unipolar depression were included in the mood group. Given symptom heterogeneity, future research should explore features associated with specific psychiatric symptoms as opposed to focusing primarily on diagnostic groups. In addition, symptom rating scales and structured diagnostic interviews should be considered to improve the reliability of symptom identification and psychiatric diagnoses, which can be impacted by patient attributes, clinical experience, and psychiatric nomenclature^72^.

Utilizing technology to assist in identifying and monitoring individuals with mental illness is a quickly advancing field. Harnessing social media platforms could be a significant step forward for psychiatry, which is limited by its reliance on mostly retrospective, self-reported data. However, how to effectively and ethically integrate patient digital data into existing clinical workflow is an important and unresolved question. Importantly, the data analyzed in the current study were obtained from consenting participants who were fully informed of the risks and benefits of participation. However, as our analyses become increasingly sophisticated and our ability to predict health outcomes improves, the potential to reveal clinical insights may motivate other groups to collect and analyze online activity without consent. Stakeholders must therefore develop standards to protect the confidentiality and the rights of this sensitive population and to avoid misuse of personal information. Interdisciplinary teams of researchers, clinicians, and patients must continue to work together to identify and solve challenges in ethics, privacy, consent, data ownership, and clinical responsibility in order to ensure that new technologies are used in the service of positive outcomes.

## Methods

Participants between the ages of 15 and 35 years old were recruited from Northwell Health’s psychiatry department. Individuals with SSD were recruited primarily from the Early Treatment Program, Northwell Health’s specialized early psychosis intervention clinic. Additional participants (*N* = 12) were recruited from three collaborating institutions located in Florida (Lauderdale Lakes) and Michigan (East Lansing and Grand Rapids). Recruitment occurred between March 2016 and December 2018. The study was approved by the Institutional Review Board (IRB) of Northwell Health (the coordinating institution) as well as local IRBs at participating sites. Written informed consent was obtained for adult participants and legal guardians of participants under 18 years of age. Assent was obtained for participating minors. Participants were eligible if they had a primary psychotic disorder or mood disorder listed in their medical records. Diagnoses were extracted from the participants’ medical records at the time of consent and were based on clinical evaluation. Healthy volunteers were approached and recruited from an existing database of eligible individuals who had already been screened for prior research projects at The Zucker Hillside Hospital and have had agreed to be re-contacted for additional research opportunities (*N* = 53). Additional healthy volunteers (*N* = 17) were recruited from a southeastern university via an online student community research recruitment site. Healthy status was determined either by the Structured Clinical Interview for DSM Disorders (SCID)^73^ conducted within the past two years, or the Psychiatric Diagnostic Screening Questionnaire (PDSQ)^74^. If clinically significant psychiatric symptoms were identified during the screening process, participants were excluded.

Participants were asked to log on to their Facebook account and request access to their Facebook archive, a feature available to all Facebook users. Participation involved a single visit during which all historical social media data were downloaded and collected. Herein, we focus on text communication sent by participants to other users in their social network, extracted from Facebook’s instant messaging services (Facebook messenger), as well as images posted by participants to their profile page. These two features were selected as they represent two of the most commonly used Facebook features, and provide rich and robust sources of data. Furthermore, Facebook messenger and images represent two largely unexplored Facebook features, as prior work utilizing Facebook data has relied nearly exclusively on status updates.

We analyzed data uploaded prior to the first psychiatric hospitalization to minimize the potential confounds of medications, hospitalizations and relapses, and receiving a formal psychiatric diagnosis on social media activity. Hospitalization date and diagnoses were drawn from the medical record. Facebook data, going back 18 months from the date of the first psychiatric hospitalization, was segmented into six temporal periods consisting of three nonoverlapping months each. Three-month blocks were chosen as they represent a period of time long enough to contain a sufficient volume of data (defined as containing at least 100 words per trimester, and for at least 50% of them, to be written in English), and also to identify symptomatic changes. The first trimester (T1) contained data from months 0 to 3 before hospitalization and the 6th trimester (T6) contained data from 15 to 18 months before hospitalization. For each participant, we averaged the data for a given trimester resulting in a vector of data. For healthy participants, the date of consent was considered to be day 0 (equivalent to hospitalization date). We also averaged data for the total 18-month period (i.e., data from all 6 trimesters) to explore the resulting data signal. Ninety (90%) of the data analyzed was generated between the years 2014–2018.

### Message processing and feature extraction

To extract linguistic features, messages posted by consenting participants were analyzed (messages received were excluded) using LIWC^75^. Given the unique nature of text in Facebook messenger, which is typically short, non-adherent to grammar or spelling rules, and often contains informal written expressions, we restricted our analysis to words. We were unable to explore sentence structure or coherence, as this would require the consideration of longer writing samples than short, unilateral Facebook posts. All LIWC features were normalized, other than “word count” which was considered to be a valid feature itself representing verbosity.

### Images processing and feature extraction

All available time-stamped photos were extracted and pre-processed using python scripts. We analyzed only the images that were in jpg format, representing output from a digital camera. We excluded all files ending in gif or png format, which are typically used for cartoons and animations. We analyzed ten features related to hue, nine features related to saturation and value, two measures related to sharpness, one for contrast, as well as the number of pixels, width, height, and brightness. Additionally, we extracted the five most used colors using the python package Colorgram. In total there were 40 features extracted per image. For features related to Hue (H), Saturation (S), and value (V), and for each pixel in the image, the red, blue, and green (RGB) representation of color model^76^ was transformed to HSV representation which aligns with how humans perceive color^76^. Nine statistical descriptors including quantiles 5, 25, 50, 75, 95, mean, variance, skewness, and kurtosis were computed for each of these distributions (HSV). We then aggregated the feature values per participant per trimester at different starting time periods (3, 6, 9, 12, and 18 months) before the first hospitalization date for patients. For all features (except hue), we aggregated using the mean of the values. Since hue is measured in degrees (angles), we used the mean of circular quantities.

Importantly, Facebook archives include data from secondary individuals who did not directly consent to participate in research, such as messages received by fellow Facebook users, as well as photos, which may contain individuals other than those consenting to research participation. We therefore explicitly disregarded messages from individuals other than those consenting to research participation using an automated process. Additionally, given that pictures posted could include identifiable information and/or secondary individuals, we only extracted abstract features such as contrast, color, and image size. The raw data were contained in a firewalled server and were never shared outside of Northwell. The processing of high-level features was implemented locally, and only those features were used for further analysis outside of the raw data server. The high-level features were always contained in HIPPA compliant servers.

### Statistical analysis

We computed the Kolmogorov–Smirnov (KS) test^77^, for each pair of conditions (HV vs. MD, HV vs. SSD, and MD vs. SSD) and for each of the computed features (described above). For multiple test correction, we performed FDR correction, using the Benjamini–Hochberg procedure^78^.

### Classification

We applied Random Forest (RF), a general-purpose classifier. Features were standardized (mean = 0 and standard deviation = 1) before being input to the classifier. We used fivefold cross-validation, leaving participants out in the test folds, which were stratified. The performance was reported as an AUC, which measures how the true positive rate (recall) and the false-positive rate trade-off. We report AUC rates that were calculated over 50 instantiations of the fivefold partition and we provide means and standard deviations. Our first objective was to evaluate whether we could distinguish between SSD, MD, and HV based on Facebook data alone. To do so, we performed a pairwise classification using the aggregated data for 18 months in the traditional cross-validation scheme. For this prediction task, each participant was represented by a single-feature vector, which indicated their state as an average overall their data across the six trimesters, which limited bias related to the frequency of posting. Our second objective was to assess if signals identified in the first trimester (when psychiatric symptoms are typically the most prominent, resulting in hospitalization) are also present in the trimesters farther away from hospitalization. We, therefore, performed classifications where the model was trained with data from the first trimester only and tested using data from the other trimesters, maintaining out the data used in the training.

Next, we evaluated a three-way classification using a general-purpose classifier, Logistic Regression (LR), with l1-norm regularization. We report the classification accuracy and F1 metric calculated over 50 instantiations of the fivefold partition. For all models, we implemented double-nested cross-validation for choosing hyperparameters, ensuring that all of the parameters required are learned within the training folds, as opposed to different iterations of training and testing cycles.

### Reporting summary

Further information on experimental design is available in the Nature Research Reporting Summary linked to this paper.

## Supplementary information

Reporting Summary Checklist

## Data Availability

The datasets analyzed during this study are not publicly available due to participant privacy and security concerns, including HIPAA regulations. The Facebook archives and health records are not redistributable to researchers other than those engaged in the IRB approved research collaborations and available from the author upon reasonable request.

## Code availability

The code implemented for this study is not publicly available due to participant privacy and security concerns, including HIPAA regulations.
