# Supplementary material for: Identifying signals associated with psychiatric illness utilizing language and images posted to Facebook
Source: NPJ Schizophr. 2020 Dec 3;6:38. doi: 10.1038/s41537-020-00125-0 (PMC7713057; doi:10.1038/s41537-020-00125-0)
Supplement: Supplementary file 1 — Reporting Summary Checklist [file 41537_2020_125_MOESM1_ESM.pdf]

nature research

Corresponding author(s): DBPR- NPJDIGITALMED-01280

Last updated by author(s): Jul 15, 2020

Reporting Summary

Nature Research wishes to improve the reproducibility of the work that we publish. This form provides structure for consistency and transparency in reporting. For further information on Nature Research policies, see Authors & Referees and the Editorial Policy Checklist.

Statistics

For all statistical analyses, confirm that the following items are present in the figure legend, table legend, main text, or Methods section.

n/a	Confirmed	
☐	☒	The exact sample size (n) for each experimental group/condition, given as a discrete number and unit of measurement
☐	☒	A statement on whether measurements were taken from distinct samples or whether the same sample was measured repeatedly
☐	☒	The statistical test(s) used AND whether they are one- or two-sided<br>*Only common tests should be described solely by name; describe more complex techniques in the Methods section.*
☐	☒	A description of all covariates tested
☐	☒	A description of any assumptions or corrections, such as tests of normality and adjustment for multiple comparisons
☐	☒	A full description of the statistical parameters including central tendency (e.g. means) or other basic estimates (e.g. regression coefficient) AND variation (e.g. standard deviation) or associated estimates of uncertainty (e.g. confidence intervals)
☐	☒	For null hypothesis testing, the test statistic (e.g. F, t, r) with confidence intervals, effect sizes, degrees of freedom and P value noted<br>*Give P values as exact values whenever suitable.*
☐	☒	For Bayesian analysis, information on the choice of priors and Markov chain Monte Carlo settings
☐	☒	For hierarchical and complex designs, identification of the appropriate level for tests and full reporting of outcomes
☐	☒	Estimates of effect sizes (e.g. Cohen's d, Pearson's r), indicating how they were calculated

Our web collection on statistics for biologists contains articles on many of the points above.

Software and code

Policy information about availability of computer code

Data collection	No software was used for data collection.
Data analysis	Linguistic Inquiry and Word Count (LIWC), a text analysis program was used for computing linguistic features. The Python package Colorgram was used to extract the 5 most used colors. We used machine learning to build classifiers and generate features.

For manuscripts utilizing custom algorithms or software that are central to the research but not yet described in published literature, software must be made available to editors/reviewers. We strongly encourage code deposition in a community repository (e.g. GitHub). See the Nature Research guidelines for submitting code & software for further information.

Data

Policy information about availability of data

All manuscripts must include a data availability statement. This statement should provide the following information, where applicable:
- Accession codes, unique identifiers, or web links for publicly available datasets
- A list of figures that have associated raw data
- A description of any restrictions on data availability

The datasets analyzed during the current study are not publicly available due to participant privacy and security concerns, including HIPAA regulations. The Facebook archives and health records are not redistributable to researchers other than those engaged in the IRB approved research collaborations.

Field-specific reporting

Please select the one below that is the best fit for your research. If you are not sure, read the appropriate sections before making your selection.

☐ Life sciences ☒ Behavioural & social sciences ☐ Ecological, evolutionary & environmental sciences

For a reference copy of the document with all sections, see nature.com/documents/nr-reporting-summary-flat.pdf

Behavioural & social sciences study design

All studies must disclose on these points even when the disclosure is negative.

Study description	This is a quantitative study that used machine learning to explore linguistic and image features associated with schizophrenia spectrum disorders (SSD) and mood disorders (MD) leading up to the first psychiatric hospitalization in participants' retrospectively collected Facebook archives.
Research sample	Participants between the ages of 15 and 35 years who had been diagnosed with SSD or MD were recruited, as well as healthy volunteers (HV). This study sample was chosen as it is representative of individuals in the early stages of a psychiatric illness who would be most likely to benefit from novel and objective early identification and monitoring strategies.
Sampling strategy	Individuals who had been diagnosed with SSD or MD were recruited through inpatient and outpatient psychiatric departments at four institutions. HVs were also recruited to serve as comparison. Eligible participants were between the ages of 15 and 35 and had an active Facebook account from which a digital archive could be extracted. Given the novel nature of this investigation, determining effect sizes a priori was not possible. However, the data included 3,404,959 Facebook messages and 142,390 Facebook Images on which to train and test the prediction models.
Data collection	Participation involved a single study visit at the time of consent during which all historical Facebook data was downloaded and collected on a study computer. Archives include all uploaded content (comments, messages, shares, likes, photos, etc.) since account creation. Clinical data including dates of hospitalizations and diagnoses were obtained through medical records. The participant and research staff were present at the time of archive collection. Researchers were not blind during data collection.
Timing	Recruitment occurred between March 2016 and December 2018.
Data exclusions	Participants were excluded (N=14) if they had a missing first hospitalization date or had unusable archive data (less than 50% of the text was in english, had insufficient Facebook data).
Non-participation	Participation involved a single study visit and no participants dropped out post consent. Approximately 55% of the individuals approached agreed to participate which is consistent with participation rates in prior research trials conducted at The Zucker Hillside Hospital involving individuals with early psychosis.
Randomization	Participants were not allocated into experimental groups.

Reporting for specific materials, systems and methods

We require information from authors about some types of materials, experimental systems and methods used in many studies. Here, indicate whether each material, system or method listed is relevant to your study. If you are not sure if a list item applies to your research, read the appropriate section before selecting a response.

Materials & experimental systems

n/a	Involved in the study	
☒	☐	Antibodies
☒	☐	Eukaryotic cell lines
☒	☐	Palaeontology
☒	☐	Animals and other organisms
☐	☒	Human research participants
☐	☒	Clinical data

Methods

n/a	Involved in the study	
☒	☐	ChIP-seq
☒	☐	Flow cytometry
☒	☐	MRI-based neuroimaging

Human research participants

Policy information about studies involving human research participants

Population characteristics	See above.
Recruitment	Participants between the ages of 15 and 35 years old who had been diagnosed with SSD or MD were screened for eligibility. Eligible participants were approached by a local research staff member and offered the opportunity to participate. Participants

	were grouped by diagnoses therefore eliminating self-selection bias. However, individuals who agreed to participate in the project may represent a biased sample of individuals who are comfortable sharing Facebook data with researchers. They are also likely to be most representative of the individuals who would agree to be monitored clinically through Facebook. Healthy volunteers who had already been screened and consented to prior studies were also recruited. If no prior health screen had been conducted, the Psychiatric Diagnostic Screening Questionnaire (PDSQ) was used to determine healthy status. Additional healthy volunteers (N=17) were recruited from a large, public university in the southeast.
Ethics oversight	The study was approved by the Institutional Review Board (IRB) of Northwell Health (the coordinating institution) as well as local IRBs at participating sites.

Note that full information on the approval of the study protocol must also be provided in the manuscript.

Clinical data

Policy information about clinical studies

All manuscripts should comply with the ICMJE guidelines for publication of clinical research and a completed CONSORT checklist must be included with all submissions.

Clinical trial registration	Not applicable. Participants were not involved in prospective intervention research.
Study protocol	The study protocol is available upon request by the authors and/or the Institutional Review Board (IRB) of Northwell Health.
Data collection	Participation involved a single study visit at the time of consent during which all historical social media data was downloaded and collected onto a local study computer. Participants from collaborating institutions sent their archives to the coordinating institution via secure IRB approved cloud-based data transmission procedures. Recruitment occurred between March 2016 and December 2018.
Outcomes	The primary outcome of interest was differentiating between psychiatric diagnoses based on facebook activity alone. This was assessed by querying participants' medical records and integrating facebook data uploaded up to 18 months in advance of the first psychiatric hospitalization. As a secondary outcome, we examined trends in linguistic and image features extracted from Facebook assocaited with escalating psychiatric symptoms closer to the date of first hospitalization.
